# Free-Range Feeding Alters Fatty Acid Composition at the *sn-2* Position of Triglycerides and Subcutaneous Fat Physicochemical Properties in Heavy Pigs

**DOI:** 10.3390/ani11102802

**Published:** 2021-09-26

**Authors:** José Segura, Ana Isabel Rey, Álvaro Olivares, María Isabel Cambero, Rosa Escudero, María Dolores Romero de Ávila, Antonio Palomo, Clemente López-Bote

**Affiliations:** 1 Departamento de Producción Animal, Facultad de Veterinaria, Universidad Complutense, Av. Puerta de Hierro s/n, 28040 Madrid, Spain; josesegu@ucm.es (J.S.); anarey@ucm.es (A.I.R.); clemente@ucm.es (C.L.-B.); 2 Sección Departamental de Tecnología de los Alimentos, Facultad de Veterinaria, Universidad Complutense, Av. Puerta de Hierro s/n, 28040 Madrid, Spain; icambero@ucm.es (M.I.C.); rmescude@ucm.es (R.E.); lolarh@vet.ucm.es (M.D.R.d.Á.); 3 Departamento de Medicina y Cirugía Animal, Facultad de Veterinaria, Universidad Complutense, Av. Puerta de Hierro s/n, 28040 Madrid, Spain; anpalomo@ucm.es

**Keywords:** swine, adipose tissue, triacylglyceride structure, positional distribution, fatty acid

## Abstract

**Simple Summary:**

Fat content and the degree of fatty acid unsaturation in meat are two major concerns for consumers. Fat concentration and its molecular structure (fatty acid positional distribution) are related to the nutritional fat value and tissue rheological properties. Changes in fat concentration and/or fatty acid profile related to modifications of dietary treatments are well described in the literature. Nevertheless, studies aimed to control fatty acid positional distribution by dietary intervention in pigs are scarce, and studies have shown that the internal *sn-2* position is highly regulated and resistant to dietary manipulation. However, this study demonstrated that heavy pigs fed on free-range with high levels of oleic acid can alter the fatty acid composition of the internal position of the triglyceride, thus affecting the nutritional value of their fat as well as their physicochemical properties.

**Abstract:**

The nutritional value of fat consumption depends on both the fatty acid composition and the positional distribution of fatty acids within the triglyceride molecule. This research studies the effect of feeding with three different diets (4% lard-enriched; 11.5% high-oleic sunflower-enriched; and extensive feeding mainly with acorns) on the composition of fatty acids in the *sn-2* position (and *sn-1,3*) of triglycerides and the textural properties of subcutaneous fat in heavy Iberian pigs (*n =* 210 castrated males). A moderate dietary enrichment with oleic acid in mixed diets did not alter the regulation of the *sn-2* position of triglyceride (69.9% and 13.9% of palmitic and oleic acids, respectively), but the extremely high intake of oleic acid in pigs fed mainly on acorns changed the proportions of palmitic and oleic acids at the *sn-2* position in the subcutaneous fat of pigs (55.0% and 27.2%, respectively). Hardness, adhesiveness, cohesiveness, gumminess, and chewiness showed the least values in EXT pigs, and the greatest values in LARD-fed barrows. SUN cohesiveness and gumminess did not differ from those fed LARD. In addition, Iberian pigs raised in free-range conditions had a more favorable nutritional lipid profile for human health compared to pigs fed conventional diets.

## 1. Introduction

Dietary fats are the main energy supply in pig diets and other monogastric animals, but their utilization for metabolic purposes depends on its absorption in the digestive system [1]. Different factors may affect their absorption and the fatty acid composition of the triglyceride structure [2]. Studies show that the external position in *sn-1* and *sn-3* in the triglyceride may impair fat absorption when compared to *sn-2*, which may affect its energy value [3]. Pork fat, compared to other animal and vegetable fats and oils, present saturated fatty acids (SFA) preferentially located in the internal *sn-2* position of the triacylglycerol molecule (TAG), reaching a concentration over 75 g SFA/100 g of total fatty acids [4]. This is considered a drawback for the nutritional value of pig meat due to the association between human diets with high SFA concentration in this position and obesity and cardiovascular diseases [5,6]. Moreover, the technological properties of pig fat are affected by the fatty acid (FA) distribution within the *sn-2* position [7,8]. Therefore, greater fat consistency has been associated with an increase in the *sn-1,3* position of SFA, which could be an interesting approach in order to control an excess of soft fat in specific pig genotypes.

Studies aimed to control the FA distribution by dietary intervention in pigs are scarce and evidence that the FA composition in the internal *sn-2* position is highly regulated and resistant to dietary manipulation [7,9], thus maintaining a narrow range of variation. Accordingly, previous studies in pigs evaluated the effects, such as dietary glycerol or saturated diets, that resulted in limited modifications at the *sn-2* position in the TAG [10]. Other dietary supplementations, such as the use of compounds that reduce the *Δ^9^*-desaturase enzyme activity, have been related to an increase in the proportion of saturated FA at the *sn-1,3* position [9]. There is no further information on the effects of the feeding with other kinds of fats or natural resources on the TAG structure. However, since a relationship between the high monounsaturated supply [11] and outdoors feeding system [12], and the increase in certain desaturases was found, their possible effect on the triglyceride structure deserves to be further explored.

Traditional feeding of Iberian pigs involves the intake of natural resources, mainly acorns and pasture. Acorns provide high-levels of monounsaturated fatty acids (MUFA), particularly oleic acid. Therefore, leading to an extremely high concentration of MUFA in pig tissues (over 55 g oleic acid/100 g total FA) [13,14]. Iberian pigs are slaughtered at high weights and studies have shown a particular lipid metabolism with increased activities of both lipogenic and desaturase enzymes [15,16]. However, there is a lack of information on how the metabolic pathways of heavy Iberian pigs fed different fat sources may affect the TAG structure. Concurrently with this line and within the industry interest of mimicking meat quality from the outdoor feeding system, monounsaturated fats have been used in the indoor dry feed. Iberian lard has been used in a traditional way as a good source of MUFA for a long time until the appearance of modified sunflower oils, that not only provide a high content of oleic acid, but also provide a low SFA in the same way as the resources that the pig consumes in free-range [14].

It was hypothesized that feeding heavy pigs with high levels of MUFA can alter the FA composition of the internal position of the TAG, thus affecting the nutritional value of their fat as well as their physicochemical properties. The objectives were to (1) study the effect of a wide range of dietary FA (saturated or monounsaturated-mixed diets indoors and monounsaturated outdoors from natural resources) given to Iberian pigs on the triglyceride structure; and (2) study the effect of the FA positional distribution changes on the rheological properties of the subcutaneous fat.

## 2. Materials and Methods

### 2.1. Experimental Design

Castrated males (*n =* 210) from the mating of purebred Iberian dams mated to Iberian × Duroc sires were selected at a live weight of 87.5 ± 5 kg and allocated at random to one of the three dietary regimens: (1) Indoors feeding with a 4%-lard-enriched diet (LARD) (8 pens; 10 pigs/pen); (2) Indoors feeding with a 11.5%-high-oleic-sunflower-enriched diet (SUN) (8 pens; 10 pigs/pen); (3) Extensive feeding in free-range conditions with acorns and grass (EXT) (*n =* 50). The indoor pigs were restricted until 87.5 kg (8 months old) and then fed *ad libitum* in the final fattening phase with the specific diet, according to the normal productive practices in Iberian pig production [17] (Table 1 and Table 2). Outdoor pigs were fed according to the traditional production practices in extensive conditions. This group is considered a reference in terms of maximum quality standards aimed at obtaining high-quality meat products. In this case, following the indications of the quality standard [17], the pigs received a longer period of restriction, in order to reach a weight of 90 kg at 12 months. The last fattening period was carried out in the Mediterranean forest with acorns and grass [13,14,18]. Food intake was not measured in free-range pigs since it is difficult to measure in outdoor conditions. However, the weight of the pigs was taken at different intervals in order to know the amount of feed to provide to indoor groups and to achieve a similar growth rate to the pigs fed under free-range conditions [12,14,18]. Water was provided *ad libitum*. At a live weight of approximately 150 ± 7 kg, 8 animals (castrated males) from each dietary treatment were slaughtered by electrical stunning and exsanguinated at a local abattoir (Mataderos Salamanca S.L., Mozárbez, Salamanca, Spain; certified under the Spanish Quality Standard for the Iberian pig R.D. 4/2014) [17]. At slaughter, back fat samples were taken at the level of the last rib and frozen under liquid N_2_. Samples were transported and kept at −80 °C until analysis (within 1 month).

### 2.2. Fat Extraction and Triacylglyceride Purification

The total lipids of the subcutaneous fat were extracted from 1 g of the outer layer [19]. TAG were purified by thin-layer chromatography (TLC) on 0.25 cm-thick silica gel plates that were developed with hexane:ethyl ether:acetic acid (75:25:1 by volume) using 30 µL of total lipids. To detect the position of the TAG, the TLC plates were sprayed with a 0.05% solution of primuline in acetone:water (8:2 by volume). Then, TAG fractions were scraped off the plates and eluted from silica with hexane:diethyl ether (95:5 by volume). In each case, the samples of purified TAG were analyzed by both gas chromatography (GC) to quantify the fatty acid profile and by lipase hydrolysis to determine the TAG structure [4].

### 2.3. Fatty Acid Profile of Subcutaneous Fat

Fatty acid methyl esters (FAME) were obtained from isolated lipids by heating the samples at 80 °C for 1 h in 3 mL of methanol:toluene:H_2_SO_4_ (88:10:2 by volume), according to the procedures outlined by Garcés and Mancha [20]. After cooling, 1 mL of hexane was added and the samples were mixed. The upper phase was recovered and the FAME were separated and quantified using a gas chromatograph (HP 6890 Series GC System, Agilent, Avondale, PA) equipped with a flame ionization detector. Separation was performed with a J&W GC Column, Innowax Polyethylene Glycol (30 m × 0.316 mm × 0.25 μm, Hewlett Packard). After injection of 5 μL, the oven temperature was raised from 170 to 210 °C at a rate of 3.5 °C/min, then to 250 °C at a rate of 7 °C/min and held constant for 1 min. The flame ionization, injector, and detector were held at 250 °C. N_2_ was used as the carrier gas, no split ratio was used, and FAME peaks were identified by comparing retention times with those of authentic standards (Sigma-Aldrich, Alcobendas, Spain).

### 2.4. Triacylglyceride Structure Analysis 

For the positional analysis of TAG *sn-2* FA, 10 mg of the purified TAG (see above) were hydrolyzed with 2 mg of pancreatic lipase in a 1 mL Tris–HCl buffer (1 M, pH 8), 0.1 mL of 22% CaCl_2_, and 0.25 mL of 0.1% deoxycholate. The reaction was stopped when approximately 60% of the TAG were hydrolyzed (1 to 2 min) by adding 0.5 mL HCl 6 N. The lipids were extracted 3 times with 1.5-mL aliquots of ethyl ether, and the reaction products were separated by TLC, as described previously. The free fatty acids (FFA) band, representing the positions 1 and 3 (*sn-1,3*), and the *sn-2*-monoacylglycerol band, representing the position 2 (*sn-2*) of TAG, were scraped off the plate and transmethylated [4]. The validity of the procedure was confirmed by comparing the FA composition of the original TAG with those remaining after the partial hydrolysis.

### 2.5. Melting Point of Subcutaneous Fat

The melting point, as determined by the slip point temperature, was performed in triplicate. Briefly, the lipids were drawn 1 cm into capillary tubes while still warm, and the tubes were stored at 4 °C overnight. Then, the tubes were placed vertically in a chilled water bath, the water temperature was increased gradually (2 °C/min), and the temperature at which the lipid began to move up the capillary tube was recorded (ISO 6321:2002) [21].

### 2.6. Texture Profile Analysis

The texture profile analysis (TPA) was carried out using a TA.XT2i SMS Stable Micro Systems Texture Analyzer (Stable Microsystems Ltd., Surrey, England) with the Texture Expert programs. Textural tests were carried out at about 22 °C. Briefly, 4 cylinders (1 cm height and 1.5 cm diameter) were prepared from each subcutaneous sample. A double compression cycle test was performed up to 50% compression of the original portion height with a 2 cm diameter aluminum cylinder probe (5 s were allowed to elapse between the two compression cycles). Force-time deformation curves were obtained with a 30 kg load cell applied at a crosshead speed of 2 mm/s. The following parameters were quantified [22]: Hardness (N) = maximum force required to compress the sample; springiness (m) = ability of the sample to recover its original form after the deforming force was removed; adhesiveness (N × s) = area under the abscissa after the first compression; cohesiveness = extent to which the sample could be deformed prior to rupture; gumminess (N) = force to disintegrate a semisolid meat sample for swallowing (hardness × cohesiveness); and chewiness (J) = work required to masticate the sample before swallowing (hardness × cohesiveness × springiness).

### 2.7. Statistical Analysis

The chemical and TPA analyses were carried out by triplicate. Response data were analyzed as a completely randomized design, two-way ANOVA, in PROC GLM of SAS v. 9.4 (SAS Institute, Inc., Cary, NC, USA, 2014) [23], with the dietary treatment as the main effect in the model. The least squares means were computed, and Duncan’s test was used to separate the means at a *p* < 0.05.

## 3. Results and Discussion

The dietary FA composition and positional distribution are important determinants in FA digestion and absorption [2]. Marked differences in FA positional distribution were found between the different fat sources of the diets (lard and high-oleic sunflower oil or acorns) (Table 2). Lard was characterized by having approximately 47.1% of oleic acid (C18:1n-9) mostly located in *sn-1,3*; 23.6% of palmitic acid (C16:0) almost fully occupying the *sn-2* position (68.9%), and similar concentrations (12-14%) of stearic and linoleic acids (C18:0 and C18:2n-6, respectively) located in a 2:1 ratio external vs. internal position. High-oleic sunflower oil and acorns had a similar concentration of C18:1n-9 (59.7%), which was located in *sn-1,3* (sunflower oil) and in *sn-2* (acorns), whereas C16:0 was fully located in *sn-1,3* of acorns and randomly distributed in high-oleic sunflower oil. In addition, C18:0 and C18:2n-6 were also located in a randomized way in high-oleic sunflower oil, and acorns had the lowest concentration of C18:0 in the *sn-1,3* position and the highest concentration of C18:2n-6, mostly found at the *sn-2* location. Previous studies reported high proportions of *sn-2* C16:0 in lard [8,24] and high *sn-2* C18:1n-9 in high-oleic sunflower oil [25]. However, there is scarce information on the triglyceride structure of fat from acorns. Mattson and Volpenhein [26] found that acorns had high proportions of *sn-2* C18:1n-9 and C18:2n-6, which agrees with the results observed in the present study, but there is no further evidence to our knowledge.

The effect of dietary treatment on the FA of TAG is presented in Table 3. Proportions of C14:0, C16:0, C18:0, as well as the total SFA and SFA/PUFA index, were less (*p* < 0.05) in subcutaneous fat of EXT pigs than those of the LARD- and SUN-fed groups (Table 3). Subcutaneous fat from pigs fed the LARD and SUN diets had greater (*p* < 0.05) proportions of palmitoleic acid (C16:1n-7), but lower proportions of eicosenoic acid (C20:1n-9) (*p* = 0.015), than the EXT pigs. Fat samples from EXT pigs had the greatest (*p* < 0.05) proportions of MUFA and UI, especially C18:1n-9, whereas the SUN-fed group had intermediate values. Similar high proportions of monounsaturated fatty acids mainly C18:1n-9 have been reported by other authors when heavy pigs were fed in extensive conditions with acorns in comparison to those fed a diet enriched with lard [27] or a fat mixture rich in monounsaturated fatty acids (lard + olive oil oleine) [18]. In addition, proportions of all PUFA, and specifically linoleic (C18:2n-6) and linolenic (C18:3n-3) acids, were greater (*p* < 0.05) in the fat from EXT pigs than fat from SUN- and LARD-fed ones. Other authors [18] reported similar results when free-range pigs were compared to the group receiving a monounsaturated-enriched diet. However, Ventanas et al. [17] reported lower PUFA in those pigs fed extensively when compared to others fed lard or a MUFA-enriched diet. Changes in the PUFA accumulation in tissues between studies may be attributed to the duration of the free-range feeding, since lower periods outdoors have resulted in higher PUFA proportions in subcutaneous fat [14]. Moreover, the PUFA proportion may be affected not only by diet, but also by other factors such as the different metabolic use by the pig, since a preferential use of this kind of fatty acids for energy supply has been reported [28].

The positional distribution of FA within the TAG molecule of pig subcutaneous fat is shown in Table 4. In contrast to other species, the *sn-2* in the TAG of pig adipose tissue was occupied mainly by C16:0, C14:0, C16:1n-7, and the SFA and SFA/PUFA index (*p* = 0.0001), as similarly described by other authors [29,30]. C18:0 was mainly esterified at the *sn-1,3* of the TAG (*p* = 0.0001), as well as C18:1n-9 (*p* = 0.0001), C18:2n-6 (*p* = 0.0001), C18:3n-3 (*p* = 0.0001), C20:1n-9, and the UI index (*p* = 0.0001). In the case of C20:0, C20:4n-6, and Σn-6/Σn-3, higher amounts were also observed in *sn-1,3* than in *sn-2* (*p* < 0.05), but no differences were detected in the total proportion of triglycerides among dietary treatments (Table 3). A similar distribution has been reported earlier in a variety of pig tissues [8,31], human milk substitutes [32,33], plasma, and milk of rats and rabbits [34], thereby indicating that FA are not randomly esterified to the glycerol hydroxyl groups in animal fats.

The increase of C18:1n-9 proportion in the SUN treatment implied a slight decrease of C16:0 (*p* = 0.0045) and SFA (*p* = 0.0001) in the *sn-1,3* position of subcutaneous fat that did not result in changes in C16:0 or SFA *sn-2* when compared to the fat samples from pigs fed lard-enriched diet. Whereas, the SUN group showed lower C18:0 accumulation in the *sn-2* position (*p* = 0.0001) when compared to the subcutaneous fat from the lard-enriched group. The decrease in the C18:0 *sn-2* proportion in fat was also observed in pigs from the EXT group when compared to fat from the LARD group in the present study. Although the change implied only around 1% variation, it has been described that the mentioned obesity and/or cardiovascular diseases related to SFA are truly related to the digested/absorbed C18:0, therefore dependent on the C18:0 concentration in the *sn-2* position [5,6]. A previous dietary intervention has also been proven to alter FA in the external *sn-1,3* position of the TAG rather than the *sn-2* location. Smith et al. [7] observed that the depressing desaturase enzyme activity increased the concentration of C18:0 located in the external *sn-1,3* position in bovine adipose tissue, but had no appreciable effect on *sn-2*. In pigs, Segura et al. [10] observed that the substitution of lard by palm oil as a dietary fat only produced slight changes in *sn-1,3*.

**Figure 1:**
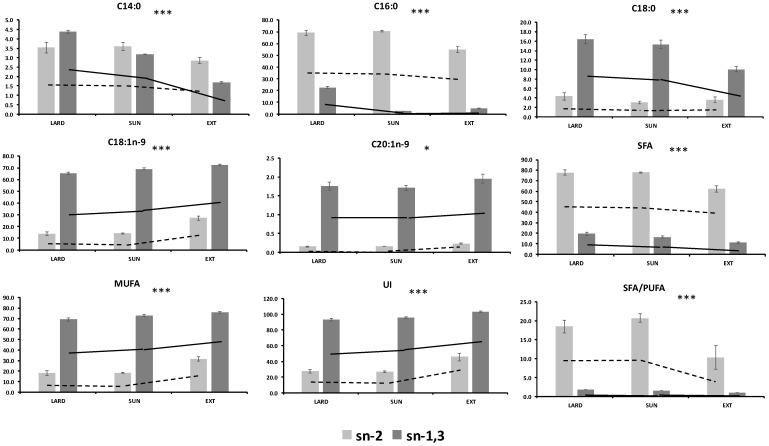
Interaction Treatment × Position of main fatty acid positional distribution. * (*p* < 0.05); *** (*p* < 0.0001) of the interaction Treatment × Position. LARD: Iberian × Duroc (IB × DR) pigs reared indoors and fed with lard as a fat source. SUN: IB × DR pigs reared indoors and fed on a diet containing high-oleic sunflower oil (115 g/kg of diet). EXT: IB × DR breed pigs free-range reared and exclusively fed on acorns and grass according to the traditional feeding system. *sn-2* (in 2-position of triglyceride), *sn*-*1,3* (average of 1- and 3-position of triglyceride). SFA (saturated fatty acids) = C14:0 + C15:0 + C16:0 + C17:0 + C18:0 + C20:0, MUFA (monounsaturated fatty acids) = C16:1n-7 + C17:1 + C18:1n-9 + C10:1n-9, UI (unsaturation index) = [(C16:1n-7 + C17:1 + C19:1n-9 + C20:1n-9) + 2 × (C18:2n-6) + 3 × (C18:3n-3 + C20:3n-6) + 4 × C20:4n-6].

The main changes by dietary manipulations on the triglyceride structure in the present study were detected in the group fed in extensive conditions mainly based on the acorns intake. Therefore, pigs fed EXT had lower C14:0, C16:0, C18:0, C20:0, and SFA (*p* < 0.05) in the *sn-2* position compared to those fed LARD or SUN diets. A decrease was also observed in *sn-1,3* of SFA in the EXT group when compared to the others but changes were of lower magnitude that those observed for the *sn-2* position. Moreover, the EXT group had an increase in *sn-2* MUFA (mainly C18:1n-9) (*p* = 0.0001) and in less magnitude in *sn-1,3* when compared to LARD or SUN. The different changes in the response to the positional distribution according to diets were confirmed by the statistically significant interaction effect (Table 4, Figure 1). Therefore, fat from EXT pigs showed a higher decrease in C14:0 and C16:0 *sn-2* and more substantial increase in C18:1n-9 *sn-2* than the other groups (*p* < 0.05). Other fatty acids (C18:2n-6, C18:3n-3, C20:0, C20:3n-6, and C20:4n-6) did not show any interaction effect depending on the dietary treatment and positional distribution.

The predominant TAG structure accumulated in pig subcutaneous fat was partly related to the TAG found in the diets. Hunter [35], Mu and Porsgaard [36], and Innis [33] reported that the FA located at the *sn-2* position suffer little alteration during digestion, with approximately 70% of the FA located in this position conserved in chylomicrons. Innis and Dyer [37] provided diets differing in the total FA composition and distribution within the TAG, and reported a limited effect of dietary treatment on liver lipid in piglets, suggesting the metabolic regulation of FA composition at the *sn-2* position. Moreover, the presence of C16:0 in *sn-2* of the dietary TAG in human infants and piglets during lactation seems to be of importance for the adequate development of the organism [33,38]. Gastric and pancreatic lipases hydrolyze FA from the external positions of the TAG. With the positional distribution of dietary fat sources used in the present experiment, the formation of FFA and 2-monoglycerides during the digestion of fat is markedly different depending on the dietary fat [30], thus the reassembly of the TAG could not be convergent.

The increase of TAG C18:2n-6 in fat from the EXT group when compared to the others, was reflected equally in both positions in the present study, thus implying a high regulation to keep a constant ratio between the external and internal position amounts of this FA. Couëdelo et al. [39] reported that the amount of linoleic acid moved from the *sn-2* position of structured TAG to the *sn-1,3* increased during absorption. Further research is needed to clarify whether this finding on the C18:2n-6 positional structure was completely due to special feeding conditions or to any other aspect of importance in outdoor production.

The effect of dietary treatments on subcutaneous fat moisture, melting point, and textural characteristics is presented in Table 5. The moisture content was higher in fat from the LARD group followed by the SUN and EXT groups. The decrease of fat moisture from pigs fed diets enriched with MUFA was also observed by other authors [40], who noted that the more saturated the fat was, the greater the moisture level resulted. The melting point, hardness, adhesiveness, cohesiveness, gumminess, and chewiness were least (*p* < 0.05) in subcutaneous fat of EXT pigs, and greatest (*p* < 0.05) in fat samples from LARD-fed barrows. Cohesiveness and gumminess of fat from pigs fed SUN-enriched diets did not differ from those fed LARD, whereas fat from LARD-fed pigs had greater (*p* < 0.05) chewiness values than the other groups.

The different responses of the melting point in subcutaneous fat from heavy pigs according to dietary fats has been reported previously [41]. In fact, the C18:0 content [42] and the relationship between MUFA and SFA [43] have been considered the best predictors of the melting point. In a more detailed study [8], in which the effect of positional distribution within the TAG molecule on selected physical properties of subcutaneous fat of dry-cured hams was analyzed, the melting point oscillations were related to the concentration and positional distribution of FA. Therefore, when C16:0 is preponderant in *sn-2*, the melting point depends largely on the FA present in *sn-1,3*. In the present study, an increase of slip point was observed when C16:0 or C18:0 were located in *sn-1,3* (LARD-fed pigs) and a decrease when C18:1n-9 was increased in this position. However, when the C18:1n-9 concentration at *sn-2* increased, the increase of C18:1n-9 and C18:2n-6 (and no C18:0) in *sn-1,3* caused the slip point to decrease in fat from the SUN-fed and EXT pigs.

Concerning fat hardness, other authors reported that higher proportions of C18:0 and lower proportions of C18:2n-6 fatty acids led to a harder fat [43]. Segura et al. [8] also observed that hardness was correlated with the FA of external positions of the TAG molecule. These authors reported positive correlations of hardness with C16:0, C18:0, and SFA in the *sn-1,3* position and negative correlations when C18:2n-6 and total PUFA occupied sn-1,3. In the present study, the lowest hardness values in fat from EXT pigs was coincident with the lowest level of C18:0 and the highest levels of C18:1n-9 and C18:2n-6 in *sn-1,3,* whereas the opposite was observed in the fat of LARD-fed pigs. Between the fat from LARD and SUN-fed pigs, the only differences in *sn-1,3* were found for C18:1n-9, total MUFA, and total SFA proportions, which could explain the changes observed on hardness between these groups.

Other textural parameters have also been related with the fatty acid profile and specific triglyceride structure. In fat of bovine kidney, Casutt et al. [44] and Nishioka and Irie [45] found that greater adhesiveness was associated with higher proportions of SFA or C18:2n-6. Furthermore, Segura et al. [8] observed that adhesiveness was dependent on the FA proportion in *sn-2*. A positive correlation was found specifically with C18:0 and C18:2n-6 *sn-2*, and was inversely proportional to the proportion of C18:1n-9 at *sn-2*. Therefore, the decreased adhesiveness of fat from EXT pigs found in the present study may be explained by the increase of C18:2n-6 and the decrease of SFA in *sn-2*.

A direct dependence of springiness and cohesiveness, and FA or triglyceride structure has not been clarified in our results. In fact, Sumena et al. [46] concluded that the contribution to the texture features, mainly cohesiveness, was the network of predominantly collagen and small quantities of elastic and reticular fibers, and not the adipocyte composition itself.

## 4. Conclusions

The *sn-2* position of the triacylglyceride in pig subcutaneous fat is highly regulated and concentrates C16:0 around 70 g/100 g fatty acids. A moderate dietary enrichment with C18:1n-9 in mixed diet produce slight changes. However, the extremely high intake of C18:1n-9 in extensively reared pigs fed mainly on acorns surpasses this regulation, thereby modifying fatty acids in the *sn-2* position. Therefore, extensive feeding increased C18:1n-9 *sn-2* and decreased C16:0 *sn-2* proportions when compared to the other dietary fats. Remarkably, the C18:0 proportion in *sn-2* decreased with dietary treatments, but the C18:2n-6 proportion and positional distribution showed a strong regulation to invariability. Consequently, Iberian pigs raised extensively would have a more favorable lipid profile from the human health perspective and distinctive fat rheological properties compared to pigs fed mixed diets containing lard or high-oleic sunflower oil. The high differences in the triglyceride structure between groups fed high-oleic diets and extensive feeding would indicate that this analysis could be an interesting tool for free-range feeding authentication.

## Figures and Tables

**Table 1:** Diet composition.

	Growing	Fattening	
Ingredients (%)	<87.5 kg	>87.5 kg	
		Lard	High-Oleic
Barley NAC 2C/11	32.6	58.5	53.4
Wheat NAC/11	40.0	30.0	30.0
Soy 47	14.3	4.50	4.40
Corn	8.90	-	-
Lard	1.00	4.00	1.20
Minerals (corrector)	3.20	3.00	3.00
High-oleic sunflower	-	0.00	8.00
Fatty acids (%)			
C16:0		24.5	18.7
C16:1		1.25	1.29
C18:0		13.2	12.5
C18:1n-9		49.8	61.7
C18:2n-6		12.5	7.10
C18:3n-3		0.43	0.48
EN (MJ/kg)	10.29	10.13	10.20
Crude protein (g/kg feed)		157.0	152.6
Crude fat (g/kg feed)		45.0	77.5
Crude fiber (g/kg feed)		50.0	57.2
Ash (g/kg feed)		52.3	63.7

**Table 2:** Triacylglyceride (*TAG*), position 2 (*sn-2*), and position 1 and/or 3 (*sn-1,3*) fatty acid composition (g/100 g quantified fatty acids) of fat sources (lard, high-oleic sunflower oil and acorns) used in the experiment.

		Lard	High-Oleic Sunflower Oil	Acorn
*TAG*	C14:0	1.07	0.871	0.070
	C16:0	23.6	18.1	15.3
	C16:1	1.92	1.95	1.88
	C18:0	13.7	12.1	2.68
	C18:1n-9	47.1	59.7	61.5
	C18:2n-6	12.1	6.78	17.9
	C18:3n-3	0.569	0.465	0.705
*sn-2*	C14:0	0.005	0.006	0.007
	C16:0	68.9	21.1	1.78
	C16:1	2.54	2.48	1.66
	C18:0	9.05	14.0	0.296
	C18:1n-9	16.1	57.2	65.3
	C18:2n-6	3.31	4.95	30.3
	C18:3n-3	0.127	0.311	0.671
*sn-1,3*	C14:0	1.60	1.30	0.101
	C16:0	0.988	16.6	22.1
	C16:1	1.61	1.68	1.99
	C18:0	16.0	11.2	3.87
	C18:1n-9	62.6	61.0	59.6
	C18:2n-6	16.4	7.69	11.7
	C18:3n-3	0.790	0.542	0.722

**Table 3:** Effect of dietary treatments on the fatty acid (g/100 g quantified fatty acids) profile of triacylglycerides of subcutaneous fat.

	LARD ^3^		SUN ^4^		EXT ^5^		MSE ^2^	*p*-Value ^1^
C14:0	1.45	^a^	1.41	^a^	1.06	^b^	0.0039	0.0001
C15:0	0.051	^ab^	0.043	^b^	0.058	^a^	0.0001	0.0139
C16:0	24.5	^a^	23.7	^a^	18.2	^b^	0.1085	0.0001
C16:1n-7	2.58	^a^	2.58	^a^	2.05	^b^	0.0192	0.0001
C17:0	0.273		0.246		0.276		0.0006	0.1203
C17:1	0.311	^a^	0.270	^b^	0.271	^b^	0.0006	0.0135
C18:0	12.4	^a^	11.2	^a^	7.87	^b^	0.2485	0.0001
C18:1n-9	48.0	^c^	50.7	^b^	57.9	^a^	0.3227	0.0001
C18:2n-6	7.71	^b^	7.13	^b^	9.44	^a^	0.0757	0.0001
C18:3n-3	0.603	^b^	0.546	^b^	0.706	^a^	0.0016	0.0001
C20:0	0.199		0.191		0.171		0.0006	0.1245
C20:1n-9	1.22	^b^	1.19	^b^	1.38	^a^	0.0040	0.0002
C20:3n-6	0.510		0.559		0.477		0.0097	0.3945
C20:4n-6	0.144		0.139		0.149		0.0001	0.2024
SFA ^6^	38.9	^a^	36.8	^a^	27.6	^b^	0.4571	0.0001
MUFA ^7^	52.1	^c^	54.8	^b^	61.6	^a^	0.3420	0.0001
PUFA ^8^	8.97	^b^	8.38	^b^	10.8	^a^	0.0837	0.0001
UI ^9^	71.5	^c^	73.0	^b^	84.6	^a^	0.7588	0.0001
Σn-6/Σn-3 ^10^	13.9		14.3		14.3		0.3259	0.2714
SFA/PUFA	4.34	^a^	4.40	^a^	2.57	^b^	0.0251	0.0001

^1^ Different letters within the same row indicate the difference between groups (*p* < 0.05). ^2^ MSE = pooled mean square error (*n =* 8). ^3^ LARD: Iberian × Duroc (IB × DR) pigs reared indoors and fed with lard as the fat source. ^4^ SUN: IB × DR pigs reared indoors and fed on a diet containing high-oleic sunflower oil. ^5^ EXT: IB × DR pigs free-range reared and exclusively fed on acorns and grass according to the traditional feeding system. ^6^ SFA (saturated fatty acids) = C14:0 + C15:0 + C16:0 + C17:0 + C18:0 + C20:0, ^7^ MUFA (monounsaturated fatty acids) = C16:1n-7 + C17:1 + C18:1n-9 + C10:1n-9, ^8^ PUFA (polyunsaturated fatty acids) = C18:2n-6 + C18:3n-3 + C20:3n-6 + C20:4n-6, ^9^ UI (unsaturation index) = [(C16:1n-7 + C17:1 + C19:1n-9 + C20:1n-9) + 2 × C18:2n-6 + 3 × (C18:3n-3 + C20:3n-6) + 4 × C20:4n-6], ^10^ Σn-6/Σn-3 = (C18:2n-6 + C20:3n-6 + C20:4n-6)/C18:3n-3.

**Table 4:** Triacylglyceride *sn-2* and *sn-1,3* fatty acid composition (g/100 g quantified fatty acids) profile of subcutaneous fat. Effect of dietary treatment (D), positional distribution (P), and the interaction (D × P).

		LARD ^3^		SUN ^4^		EXT ^5^		MSE ^2^	*p*-Value ^1^		
									D	P	D × P
C14:0	*sn-2*	3.54	^a^	3.61	^a^	2.85	^b^	0.0270	0.0001	0.0001	0.0010
	*sn-1,3*	0.439	^a^	0.318	^b^	0.168	^c^				
C15:0	*sn-2*	0.146	^a^	0.117	^b^	0.150	^a^	0.0003	0.0281	0.0001	0.2167
	*sn-1,3*	0.016		0.007		0.013					
C16:0	*sn-2*	69.2	^a^	70.7	^a^	55.0	^b^	2.4494	0.0001	0.0001	0.0001
	*sn-1,3*	2.26	^a^	1.23	^b^	0.460	^c^				
C16:1n-7	*sn-2*	3.76		3.76		3.69		0.1861	0.0459	0.0001	0.1163
	*sn-1,3*	1.99	^a^	2.00	^a^	1.23	^b^				
C17:0	*sn-2*	0.425	^b^	0.367	^b^	0.507	^a^	0.0020	0.0335	0.0001	0.0009
	*sn-1,3*	0.197	^a^	0.185	^ab^	0.160	^b^				
C17:1	*sn-2*	0.356	^b^	0.321	^b^	0.417	^a^	0.0022	0.1898	0.0001	0.0008
	*sn-1,3*	0.289	^a^	0.244	^ab^	0.198	^b^				
C18:0	*sn-2*	4.31	^a^	3.01	^b^	3.56	^b^	0.5657	0.0001	0.0001	0.0001
	*sn-1,3*	16.4	^a^	15.3	^a^	10.0	^b^				
C18:1n-9	*sn-2*	13.7	^b^	14.0	^b^	27.2	^a^	1.5760	0.0001	0.0001	0.0001
	*sn-1,3*	65.2	^c^	68.1	^b^	72.7	^a^				
C18:2n-6	*sn-2*	3.33	^b^	2.82	^b^	5.23	^a^	0.3687	0.0001	0.0001	0.8371
	*sn-1,3*	9.80	^b^	9.29	^b^	11.4	^a^				
C18:3n-3	*sn-2*	0.333	^b^	0.297	^b^	0.528	^a^	0.0064	0.0001	0.0001	0.1407
	*sn-1,3*	0.727	^b^	0.670	^b^	0.795	^a^				
C20:0	*sn-2*	0.142	^a^	0.130	^a^	0.090	^b^	0.0015	0.1195	0.0001	0.6713
	*sn-1,3*	0.227		0.222		0.211					
C20:1n-9	*sn-2*	0.147	^b^	0.156	^b^	0.220	^a^	0.0054	0.0001	0.0001	0.0309
	*sn-1,3*	1.76	^b^	1.71	^b^	1.95	^a^				
C20:3n-6	*sn-2*	0.456		0.542		0.471		0.0393	0.6832	0.5952	0.9002
	*sn-1,3*	0.537		0.567		0.481					
C20:4n-6	*sn-2*	0.105		0.107		0.135		0.0010	0.5565	0.0012	0.3539
	*sn-1,3*	0.164		0.155		0.156					
SFA ^6^	*sn-2*	77.8	^a^	78.0	^a^	62.1	^b^	3.9942	0.0001	0.0001	0.0001
	*sn-1,3*	19.6	^a^	16.3	^b^	11.0	^c^				
MUFA ^7^	*sn-2*	18.0	^b^	18.3	^b^	31.5	^a^	2.6230	0.0001	0.0001	0.0001
	*sn-1,3*	69.2	^c^	73.0	^b^	76.1	^a^				
PUFA ^8^	*sn-2*	4.23	^b^	3.76	^b^	6.36	^a^	0.3805	0.0001	0.0001	0.5854
	*sn-1,3*	11.2	^b^	10.7	^b^	12.9	^a^				
UI ^9^	*sn-2*	27.4	^b^	26.9	^b^	45.5	^a^	6.1958	0.0001	0.0001	0.0001
	*sn-1,3*	93.3	^b^	95.9	^b^	103.4	^a^				
Σn-6/Σn-3 ^10^	*sn-2*	11.7		11.7		11.4		1.2720	0.8128	0.0001	0.4744
	*sn-1,3*	14.5		14.9		15.3					
SFA/PUFA	*sn-2*	18.5	^a^	20.8	^a^	10.3	^b^	2.5737	0.0001	0.0001	0.0001
	*sn-1,3*	1.74	^a^	1.53	^b^	0.859	^c^				

^1^ Different letters within the same row indicate the difference between groups (*p* < 0.05).^2^ MSE = pooled mean square error (*n =* 8). ^3^ LARD: Iberian × Duroc (IB × DR) pigs reared indoors and fed with lard as the fat source. ^4^ SUN: IB × DR pigs reared indoors and fed on a diet containing high-oleic sunflower oil. ^5^ EXT: IB × DR pigs free-range reared and exclusively fed on acorns and grass according to the traditional feeding system. ^6^ SFA (saturated fatty acids) = C14:0 + C15:0 + C16:0 + C17:0 + C18:0 + C20:0, ^7^ MUFA (monounsaturated fatty acids) = C16:1n-7 + C17:1 + C18:1n-9 + C10:1n-9, ^8^ PUFA (polyunsaturated fatty acids) = C18:2n-6 + C18:3n-3 + C20:3n-6 + C20:4n-6, ^9^ UI (unsaturation index) = [(C16:1n-7 + C17:1 + C19:1n-9 + C20:1n-9) + 2 × C18:2n-6 + 3 × (C18:3n-3 + C20:3n-6) + 4 × C20:4n-6], ^10^ Σn-6/Σn-3 = (C18:2n-6 + C20:3n-6 + C20:4n-6)/C18:3n-3.

**Table 5:** Effect of dietary treatments on moisture, melting point, and textural parameters of pig subcutaneous fat ^1^.

	LARD		SUN		EXT		MSE ^3^	*p*-Value ^2^
Moisture (%)	6.5	^a^	5.7	^b^	4.80	^c^	0.16	0.0001
Melting Point (°C)	30.5	^a^	29.4	^b^	26.4	^c^	0.19	0.0001
Hardness (N)	44.1	^a^	40.6	^b^	32.9	^c^	1.14	0.0039
Adhesiveness (N × s)	−0.41	^a^	-0.27	^b^	-0.07	^c^	0.04	0.0001
Springiness (×10^−3^) (m)	1.11	^a^	0.47	^b^	0.67	^ab^	0.20	0.1001
Cohesiveness	0.56	^a^	0.59	^a^	0.50	^b^	0.02	0.0225
Gumminess (N)	25.0	^a^	24.0	^a^	16.6	^b^	1.67	0.0038
Chewiness (×10^−2^) (J)	2.82	^a^	1.11	^b^	1.09	^b^	0.49	0.0400

^1^ LARD: Iberian × Duroc (IB × DR) pigs reared indoors and fed with lard as a fat source. SUN: IB × DR pigs reared indoors and fed on a diet containing high-oleic sunflower oil. EXT: IB × DR breed pigs free-range reared and exclusively fed on acorns and grass according to the traditional feeding system. ^2^ Different letters within the same row indicate the difference between groups (*p* < 0.05). ^3^ MSE = pooled mean square error (*n =* 8).

## Data Availability

Not applicable.
